# Sociality in Diverse Societies: A Regional Analysis Across European Countries

**DOI:** 10.1007/s11205-012-0021-0

**Published:** 2012-03-17

**Authors:** Ferry Koster

**Affiliations:** 1 Department of Sociology, Faculty of Social Sciences, Erasmus University Rotterdam, Burgemeester Oudlaan 50, PO Box 1738, 3000 DR Rotterdam, The Netherlands; 2 Amsterdam Institute for Advanced Labour Studies, University of Amsterdam, Amsterdam, The Netherlands; 3 Leiden Law School, University of Leiden, Leiden, The Netherlands

**Keywords:** Diversity, Fractionalization, Sociality, Trust, Welfare state, Empirical comparison

## Abstract

For a long time, researchers investigate the impact of diversity on society. To measure diversity, either archival data at the national level of census data at the neighborhood level, within a single country are used. Both approaches are limited. The first approach does not allow to investigate variation in diversity within countries and the second approach misses the possibility to investigate cross national differences. The present study aims at bringing these two approaches closer together by constructing diversity measures based on the European Social Survey (ESS). The ESS is collected every 2 years since 2002 and includes individual level data that allow replicating earlier measures of ethnic, linguistic, and religious diversity for 30 European countries. Furthermore, since respondents are asked to indicate in what region they live, measured with the Nomenclature of Territorial Units for Statistics classification, it is possible to construct disaggregated measures. Comparing the new indicators with existing diversity scores leads to the following conclusions. First, the new and old measures are strongly correlated at the national level. Secondly, investigating the relationship between diversity and different kinds of sociality (interpersonal trust, institutional trust, and support for government redistribution) shows that regional diversity is more strongly related to them than diversity at the national level.

## Introduction

Studies examining the impact of diversity have a long history in the social sciences (see for classical examples: Smith 1960; Blau 1977). Although diversity can refer to all kinds of differences between people, such as gender, age, occupation, socioeconomic status, and lifestyles, there has been particular interest in ethnic, linguistic, and religious dimensions (Fearon 2003; Alesina et al. 2003). Empirical studies of societal diversity differ with regard to their level of analysis. International comparative studies measure diversity at the national level using archival data and examine how it relates to a country’s social policy (Alesina and Glaeser 2004) or social capital and citizenship behavior at the individual level (Anderson and Paskeviciute 2006; Gesthuizen et al. 2009). Single country studies investigate diversity at lower levels of aggregation using census data, relating it to individual levels of trust and social cohesion, for example (Leigh 2006; Putnam 2007; Tolsma et al. 2009). The international comparative studies provide valuable information about cross national differences, but their measures of diversity do not capture potential variation in diversity within countries. As such, these measures may for instance overlook the possibility that diverse countries can consist of homogeneous regions. Additionally, it may be questioned whether national level indices measure the relevant parts of people’s social context and their preferences, attitudes and behavior. The single country studies, on the other hand, do rely on more fine-grained indicators of diversity, but they do not allow for a comparison of the effects of diversity across countries. Therefore, they do not provide information about how the impact of diversity differs depending on cross national variation in institutions and economic circumstances.

The present study aims at bridging the gap between these two empirical approaches by investigating to what extent individual attitudes relate to regional diversity across European countries. This article consists of two parts. First, new indicators of diversity are constructed. Instead of using archival or census data, individual responses on an international survey are examined to investigate the diversity of countries and regions. For that purpose, this paper investigates data from the European Social Survey (ESS), which have been collected every 2 years since the first round in 2002. Besides that respondents are asked about their opinions, attitudes and behavior towards different issues, the ESS includes information about their country of birth, the language they usually speak, and their religious denomination. These responses reflect the ethnic, linguistic, and religious fractionalization investigated by Alesina et al. (2003) and others. In the remainder of this article, the ESS based measures are referred to as “regional diversity” to distinguish them from the fractionalization scores, which will be referred to as “national diversity”. The newly created measures are compared with the existing measures. In this part of the analysis, the regional data are aggregated to the national level since the existing measures are also at that level. In addition to that it is investigated whether diversity varies within countries, based on the region in which the respondent lives. These regions are classified using the Nomenclature of Territorial Units for Statistics (NUTS) (Eurostat 2007). The NUTS regions provide a harmonized system of classification across EU Member States and a similar classification is developed for other countries within the European Economic Area (EEA) and Switzerland. The NUTS classification comprises a hierarchical system. The available data allow investigating variation within countries at the NUTS level 1 (ranging in size from 3 to 7 million people). Previous studies included the regional level by investigating issues such as divorce (Kalmijn and Uunk 2007) and poverty (Callens and Croux 2009) and they are also a main point of reference in the European System of Social Indicators (Noll 2002). The present study investigates diversity across 123 regions in 30 countries.

The second part of the article examines to what extent such within country variation matters for understanding different kinds of sociality, namely interpersonal trust, trust in institutions, and support for government redistribution. These analyses are conducted by pooling the information from the first 4 rounds of the ESS, generating information about 173,597 respondents.

## Measuring Diversity

### Prior Measures and Datasets

The underlying logic of constructing measures of social structures is that people have a certain position within a given social space, be it a neighborhood, a workplace, a region, or a country (Blau 1977; Allison 1978; Esteban and Ray 1994). Summarizing these positions generates information about the level of similarity within these spaces, which in the aggregate are believed to be related to all kinds of outcomes such as economic prosperity and trust. The gini coefficient is a prime example of these summary scores indicating the income distribution within a country. When the summary score is based on a continuous variable it is possible to construct an index measuring inequality similar to the gini coefficient. Nevertheless, ethnic, linguistic and religious diversity reflect categorical differences in society. When there are two categories, for example if a distinction is made between foreigners and non-foreigners, providing the mean may suffice to represent the level of dissimilarity, but when there are more than two categories a different methodological strategy is required. Researchers from different scientific backgrounds suggest similar diversity scores based on the following formula.

$$ D = 1 - \sum\limits_{i = 1}^{N} {p_{i}^{2} } $$

where *p* proportion of individuals per category, *N* number of categories.

A score of 0 indicates a perfectly homogenous population and 1 a perfectly heterogeneous one. The index indicates the probability that two randomly selected individuals from the population belong to different groups. With regard to the definition of what constitutes group differences, ethnic divisions have proven to be particularly hard to define and measure. Reviews of the literature show that ethnic background is a problematic concept that is poorly defined in many papers and many researcher do not provide a definition at all (Isajiw 1974; Cohen 1978). Definitions of “ethnicity” or “ethnic group” refer to the notion that members of the group are aware of their membership, have a common origin and culture, or believe that others think of them as having these attributes (Yinger 1985) and that groups can be differentiated by color, language and religion (Horowitz 1985). Descriptions of ethnicity include both factors that can be measured (relatively) objectively, such as nationality, language, and country of origin, and subjective evaluations like ethnic identity and group awareness. The subjective part of ethnicity is not included in datasets measuring diversity. The measures developed in this study follow these measures and therefore also focus on the objective rather than the subjective aspects of ethnicity. The level of ethno-linguistic fractionalization (ELF) of a country has been a prominent variable for a long time and is investigated in a number of studies (Taylor and Hudson 1972; Fearon 2003; Fearon and Laitin 2003; Montalvo and Reynal-Querol 2005). However, it has been argued that the ELF variable may have an important weakness since it combines linguistic and ethnic heterogeneity, whereas these two variables do not necessarily measure the same construct. In response to that criticism, researchers have distinguished ethnic and linguistic dimensions of diversity, while extending it with indicators of religious diversity (Hansmann and Quigley 1982; Alesina et al. 2003).

### Using Survey Data to Investigate Diversity

International comparative surveys offer a unique opportunity for constructing indicators of diversity. For the present study, data from the European Social Survey (ESS) are examined for the following reasons. First, this dataset allows constructing diversity scores and to compare them empirically across the majority of European countries with existing measures of diversity. This research strategy provides an external validation of the constructed variables. Secondly, the ESS includes a variable indicating the region where people live, thus allowing for the construction of indicators at disaggregated levels.

The variables of interested are country of origin which is measured with the question “In which country were you born?”, language which is asked with the question “What language or languages do you speak most often at home?” and religion measured with the item “Do you consider yourself as belonging to any particular religion or denomination” and if the respondent answers yes, the follow up question is asked: “Which one?”. These individual answers are aggregated to the country level to construct the national level indicators and to the NUTS level 1 level to construct the regional indictors. It should be noted that the NUTS level 1 regions may refer to somewhat different units across countries as some reflect preexisting administrative units, while on the other hand they consist of a regrouping of lower level units. As a result, the actual meaning may differ somewhat between countries. Nevertheless, since the main goal of this classification scheme is to enable harmonization of data (Eurostat 2007), it is assumed that the regions can be compared. This results in a mean value for the categories within a country or a region. The scores for regional diversity are calculated with the diversity index mentioned earlier to construct regional level measures of diversity in origin, and linguistic and religious diversity.

To date, four rounds of the ESS are released, starting from 2002 every 2 years a new round has become available. In total 33 countries participated in these four rounds. However, the number of times that countries participated varies since not every country participated in every round. There are 15 countries for which data are available for all four rounds, 7 countries participated three times, 7 other countries participated two times, and in 4 countries data have been gathered only once. Round 1 includes 22 countries, in Round 2, 26 countries participated, Round 3 includes 23 countries, and Round 4 consists of 28 countries. To get data from a broad range of countries, the average scores are computed for each country across the four rounds of the ESS. Table 1 provides an overview of the ESS Rounds, the countries, and the number of respondents. Appendix Table 10 shows the number of respondents and the ESS heterogeneity scores for each country disaggregated to the NUTS level 1 region. For 11 countries it holds that the country level is the same as the NUTS level. This means that the diversity measures cannot be disaggregated to a lower level based on this classification scheme. The level of intra-country diversity of the remaining 19 countries can be further examined as they fall into a number of different NUTS level 1 regions. The number of regions differs from 2 (Bulgaria and Finland) to 16 (Germany).

**Table 1:** Number of respondents per country and per ESS round

	Round 1	Round 2	Round 3	Round 4	Total
Austria	2,257	2,256	2,405	–	6,918
Belgium	1,899	1,778	1,798	1,760	7,235
Bulgaria	–	–	1,400	2,230	3,630
Croatia	–	–	–	1,484	1,484
Cyprus	–	–	995	1,215	2,210
Czech Republic	1,360	3,026	–	2,018	6,404
Denmark	1,506	1,487	1,505	1,610	6,108
Estonia	–	1,989	1,517	1,661	5,167
Finland	2,000	2,022	1,896	2,195	8,113
France	1,503	1,806	1,986	2,073	7,368
Germany	2,919	2,870	2,916	2,751	11,456
Greece	2,566	2,406	–	2,072	7,044
Hungary	1,685	1,498	1,518	1,544	6,245
Iceland	–	579	–	–	579
Ireland	2,046	2,286	1,800	–	6,132
Italy	1,207	1,529	–	–	2,736
Latvia	–	–	–	1,980	1,980
Luxembourg	1,552	1,635	–	–	3,187
Netherlands	2,364	1,881	1,889	1,778	7,912
Norway	2,036	1,760	1,750	1,549	7,095
Poland	2,110	1,716	1,721	1,619	7,166
Portugal	1,511	2,052	2,222	2,367	8,152
Romania	–	–	–	2,146	2,146
Spain	1,729	1,663	1,876	2,576	7,844
Slovakia	–	1,512	1,766	1,810	5,088
Slovenia	1,519	1,442	1,476	1,286	5,723
Sweden	1,999	1,948	1,927	1,830	7,704
Switzerland	2,040	2,141	1,804	1,819	7,804
Turkey	–	1,856	–	2,416	4,272
United Kingdom	2,052	1,897	2,394	2,352	8,695
Total	39,860	47,035	38,561	48,141	173,597

*Source:* European Social Survey

### Comparing Existing with New Measures of Diversity

#### Representation of Foreigners in the ESS

Even though the ESS aims at including a representative sample of citizens of each country, the question can be asked to what extent the data reflect the characteristics of the national population to allow for the construction of aggregated measures. To begin with, Appendix Table 10 shows the missing values for each of the three variables per region. It turns out that the number of missing values is low. On average far below 0.01 % is missing. The question about religion or denomination seems to be answered somewhat less often than the country of origin and the language question. Nevertheless, the only real outlier here is Slovenia with around 8 percent missing on this variable. Next to investigating the number of missings, the ESS data are compared to an external source, namely the Eurostat database providing information about the number of foreigners in a country (defined as non-nationals, meaning persons who are not citizens of their country of residence). These data are available for 25 of the 33 countries for the period between 2000 and 2008. Table 2 summarizes the outcomes. On average, the foreign population constitutes 7 % of the total population of the 25 countries according to the Eurostat dataset. The Eurostat data are compared to the ESS indicator asking about people’s country of origin. Overall, the number of foreign born people in the ESS is slightly higher than the proportion of foreigners reported by Eurostat (0.08 versus 0.07). As Table 2 shows, in most countries these numbers are somewhat higher in the ESS than in the Eurostat database. On the other hand, the differences between this ESS indicator and the Eurostat measures are considerable for countries like Luxembourg and Latvia (both 9 percent difference). Furthermore, the Eurostat and the ESS indicators turn out to be strongly related (r = 0.924; *p* < 0.001).

**Table 2:** Comparison of Eurostat and European Social Survey (ESS)

	% Foreigners (Eurostat)	% Foreign born (ESS)	Difference
Austria	0.09	0.08	0.01
Belgium	0.08	0.09	−0.01
Bulgaria	0.00	0.01	−0.01
Cyprus	0.11	0.07	0.04
Czech Republic	0.02	0.03	−0.01
Denmark	0.05	0.06	−0.01
Estonia	0.19	0.20	−0.01
Finland	0.02	0.03	−0.01
France	0.06	0.09	−0.03
Germany	0.09	0.08	0.01
Greece	0.08	0.09	−0.01
Hungary	0.01	0.02	−0.01
Ireland	0.06	0.09	−0.03
Italy	0.03	0.02	0.01
Latvia	0.23	0.14	0.09
Luxembourg	0.39	0.30	0.09
Netherlands	0.04	0.08	−0.04
Poland	0.00	0.01	−0.01
Portugal	0.02	0.06	−0.04
Romania	0.00	0.01	−0.01
Slovakia	0.00	0.03	−0.03
Slovenia	0.02	0.08	−0.06
Spain	0.05	0.07	−0.02
Sweden	0.05	0.11	−0.06
United Kingdom	0.05	0.10	−0.05
Total	0.07	0.08	−0.01

*Sources:* Eurostat 2000–2008, European Social Survey 1–4

Croatia, Iceland, Norway, Switzerland and Turkey are missing because they are not included in Eurostat

These comparisons show that the ESS includes a sizable number of foreigners, that the necessary information is provided by most respondents, and that the proportion of foreigners in the ESS is strongly related to that of an external source (Eurostat), but also that it should be realized that in some countries may slightly underestimate the number of actual foreigners (non-nationals) and that this particularly holds for some of the outliers in the dataset.

#### Comparing Regional Diversity with National Diversity

Table 3 provides an overview of the national diversity measures (the fractionalization data of Alesina et al. 2003) with the regional diversity measures based on the ESS data (aggregated to the national level to enable comparisons). The average scores of the indicators that are constructed with the ESS are consistently lower than the national diversity scores. While the ethnic diversity of the European countries is 0.25, their average diversity in origin is 0.15 for the regional diversity measure based on the country of origin of ESS respondents. The mean level of linguistic diversity at the national level is also 0.25 in the existing dataset, whereas the ESS reports a mean of 0.18. And, the level of religious diversity is 0.37 at the national level compared to 0.26 based on the religious diversity at the regional level. Again there are some noteworthy country similarities and differences, as can be read in Table 4. For a number of countries it holds that the level of national diversity and the ESS based mean level of regional diversity is quite similar. And for some countries the measures are exactly the same: Norway, Portugal, and Sweden have a difference of zero. There are 18 countries in the dataset for which the difference between the national level ethnic diversity and the ESS diversity measure based on the country of origin measure of the ESS. On the other hand, there are countries with a difference around 0.30 (Croatia and Turkey) and it also turns out that for some countries (Sweden and Sweden for example) the level of regional diversity in origin computed with the ESS is higher compared to the national level of ethnic diversity.

**Table 3:** National and regional diversity per country

	National diversity			Regional diversity		
	Ethnic	Linguistic	Religious	Origin	Linguistic	Religious
Austria	0.11	0.15	0.41	0.15	0.06	0.21
Belgium	0.56	0.54	0.21	0.16	0.51	0.22
Bulgaria	0.40	0.30	0.60	0.02	0.25	0.36
Croatia	0.37	0.08	0.04	0.16	0.01	0.14
Cyprus	0.09	0.40	0.40	0.12	0.04	0.03
Czech Republic	0.32	0.32	0.66	0.06	0.02	0.25
Denmark	0.08	0.10	0.23	0.11	0.04	0.13
Estonia	0.51	0.49	0.50	0.33	0.43	0.54
Finland	0.13	0.14	0.25	0.04	0.12	0.07
France	0.10	0.12	0.40	0.17	0.10	0.24
Germany	0.17	0.16	0.66	0.15	0.07	0.57
Greece	0.16	0.03	0.15	0.16	0.07	0.07
Hungary	0.15	0.03	0.52	0.04	0.01	0.43
Iceland	0.08	0.08	0.19	0.06	0.01	0.16
Ireland	0.12	0.03	0.16	0.17	0.05	0.10
Italy	0.11	0.11	0.30	0.04	0.24	0.03
Latvia	0.59	0.58	0.56	0.25	0.42	0.70
Luxembourg	0.53	0.64	0.09	0.50	0.50	0.43
Netherlands	0.11	0.51	0.72	0.16	0.09	0.62
Norway	0.06	0.07	0.20	0.12	0.09	0.20
Poland	0.12	0.05	0.17	0.03	0.00	0.03
Portugal	0.05	0.02	0.14	0.11	0.02	0.07
Romania	0.31	0.17	0.24	0.01	0.14	0.22
Slovakia	0.25	0.26	0.57	0.05	0.20	0.32
Slovenia	0.22	0.22	0.29	0.15	0.04	0.13
Spain	0.42	0.41	0.45	0.14	0.25	0.10
Sweden	0.06	0.20	0.23	0.20	0.10	0.28
Switzerland	0.53	0.54	0.61	0.35	0.53	0.58
Turkey	0.32	0.22	0.00	0.02	0.23	0.03
United Kingdom	0.12	0.05	0.69	0.18	0.07	0.58
Total	0.25	0.25	0.37	0.15	0.18	0.26

*Sources:* Alesina et al. (2003) and European Social Survey 1–4

Regional diversity aggregated to the national level

**Table 4:** Differences between national and regional diversity per country

	Ethnic/origin	Linguistic	Religious
Austria	−0.04	0.09	0.20
Belgium	0.40	0.03	−0.01
Bulgaria	0.38	0.05	0.24
Croatia	0.21	0.07	−0.10
Cyprus	−0.03	0.36	0.37
Czech Republic	0.26	0.30	0.41
Denmark	−0.03	0.06	0.10
Estonia	0.18	0.06	−0.04
Finland	0.09	0.02	0.18
France	−0.07	0.02	0.16
Germany	0.02	0.09	0.09
Greece	0.00	−0.04	0.08
Hungary	0.11	0.02	0.09
Iceland	0.02	0.07	0.03
Ireland	−0.05	−0.02	0.06
Italy	0.07	−0.13	0.27
Latvia	0.34	0.16	−0.14
Luxembourg	0.03	0.14	−0.34
Netherlands	−0.05	0.42	0.10
Norway	−0.06	−0.02	0.00
Poland	0.09	0.05	0.14
Portugal	−0.06	0.00	0.07
Romania	0.30	0.03	0.02
Russia	0.14	0.15	0.22
Slovenia	0.07	0.18	0.16
Slovakia	0.20	0.06	0.25
Spain	0.28	0.16	0.35
Sweden	−0.14	0.10	−0.05
Switzerland	0.18	0.01	0.03
Turkey	0.30	−0.01	−0.03
United Kingdom	−0.06	−0.02	0.11
Total	0.09	0.07	0.10

*Sources:* Alesina et al. (2003) and European Social Survey 1–4

Regional diversity aggregated to the national level

Comparing the two datasets on the linguistic dimension of diversity shows that the existing national level diversity measures the diversity scores based on the ESS are closer to each other than the ethnicity-related diversity measures. The indicators fall into a range of 0.10 for 21 of the 30 countries. The difference between the level of linguistic diversity at the national level and linguistic diversity at the regional level is particularly large in Czech Republic (a difference of 0.30), Cyprus (a difference of 0.36), and the Netherlands (a difference of 0.42). The linguistic national level diversity score of Italy is lower than the ESS based measure of linguistic diversity (a difference of 0.13). With regard to the level of religious diversity, the two types of indicators show the following similarities and differences. There are 18 countries with a difference within the plus and minus 0.10 range and five countries have a difference that is larger than 0.30, Czech Republic (0.41), Cyprus (0.37), Spain (0.35), and Luxembourg (−0.34).

The relationships between the different indicators of diversity are presented in Table 5. Ethnic diversity at the national level correlates with regional level diversity in origin aggregated to the national level (r = 0.455), the two indicators measuring linguistic diversity are positively related (r = 0.818) and the same holds for religious diversity (r = 0.695). Table 5 also shows the interrelations between the different indicators. In accordance with earlier findings, the correlation coefficients national level ethnic and linguistic diversity are positively related to each other (r = 0.770) and are less strongly related to the religious diversity of a country (r = 0.177 for ethnic diversity and r = 0.346 for linguistic diversity). The patterns are somewhat similar when the ESS measures are investigated. The diversity in origin measure is positively related to linguistic diversity (r = 0.567 and r = 0.652). The correlations between religious diversity on the one hand and diversity in origin and linguistic diversity on the other are different than those reported for the national level diversity measures: diversity in origin and religious diversity at the regional level are positively related (r = 0.462 and r = 0.472), while linguistic and religious diversity have the lowest correlations (r = 0.419). Furthermore, Table 5 provides insight into how the diversity scores from the different sources (e.g. the existing national level measures and the newly created regional level measures aggregated to the national level) are related to each other. With regard to ethnic diversity at the national level it is worth mentioning that this indicator of diversity is most strongly related to the linguistic diversity measured with the ESS (r = 0.821). Moreover, linguistic diversity at the national level is also strongly related to the ESS based indicator of regional linguistic diversity (r = 0.818). A possible interpretation of this result, in combination with the strong relation between ethnic and linguistic diversity at the national level and that there is a less strong relationship between the ethnic diversity at the national level and the regional diversity in origin, is that despite the effort to distinguish ethnic and linguistic diversity in earlier research, the indicator of national level ethnic diversity used in prior studies mainly reflects the linguistic diversity of a country.

**Table 5:** Correlation coefficients between national and regional diversity

	National diversity			Regional diversity	
	Ethnic	Linguistic	Religious	Origin	Linguistic
National diversity					
Ethnic					
Linguistic	0.770**				
Religious	0.177	0.346*			
Regional diversity					
Origin	0.455**	0.628**	0.053		
Linguistic	0.821**	0.818**	0.160	0.652**	
Religious	0.414*	0.506**	0.695**	0.472**	0.419*

*Sources:* Alesina et al. (2003) and European Social Survey 1–4

Regional diversity aggregated to the national level. N = 30 countries

** *p* < 0.01; * *p* < 0.05

The results regarding religious diversity are clear-cut. This variable is not related to any of the diversity scores based on the ESS except for religious diversity (r = 0.695), suggesting that these indicators essentially measure similar aspects of the countries investigated in this sample.

The comparison of the ESS data with two external sources supports the following conclusions. First, on average, the ESS includes shares of foreigners that are quite similar to the figures provided by Eurostat. However, since for some countries it holds that the ESS shares of foreigners deviate considerably from the Eurostat numbers, it can be considered to further explore whether this has an effect on the resulting indices. In empirical analyses, it is possible to investigate how severe inclusion or exclusion of these countries affects the outcomes by conducting a sensitivity analysis. Secondly, comparing different indicators measuring societal diversity, leads to the conclusion that the ESS based scores are consistently lower than the diversity scores provided in previous research, which were taken as a point of reference. With regard to this outcome, it cannot simply be stated that the ESS underestimates the level of diversity as it is not clear what the true level of diversity is in the countries included in the analysis and it may as well be concluded that the national level diversity scores overestimate the actual level. There may be a number of reasons for these differences. First, the ESS may include too little shares of people from different backgrounds, thus lacking the ability to capture the diversity to the fullest. Secondly, as noted above, related but different dimensions of diversity may be measured in both datasets. In particular, this remark relates to the close relationship between ethnic and linguistic diversity. And, thirdly, part of the difference can result from the difference between using a more objective source (archival data measuring national level diversity) and self-reports involving a certain level of subjectivity.

Given the differences between the ESS indicators and the external sources, it is acknowledged that ESS data are not completely the same as the Eurostat and existing diversity data at the national level. However, a focus on the similarities between the measures, by investigating differences and correlations at the country level, also shows that the indicators are interrelated and thus provide evidence that the measures at least reflect quite some overlap between the two data sources.

### Regional Diversity Measures

So far, the focus was on cross national variation in diversity, a topic that has received quite some attention in prior research, whereas other studies have focused on diversity at lower levels of aggregation. A combination of these two strategies, investigating diversity at regional levels across a sample of countries, is not available yet. With the ESS data this can be investigated as respondents are asked about their region of residence.

The data in Appendix Table 10 provide some evidence that NUTS level 1 regions within countries differ with regard to their level of diversity measured with the ESS indicators. Table 6 investigates these differences more closely. Table 6 reports the country means, standard deviations (based on the NUTS level 1 regions) and that range of regional diversity (the difference between the minimum and the maximum value of the NUTS level 1 region within a country).

**Table 6:** Descriptives of regional diversity per country

	Diversity in origin		Linguistic diversity		Religious diversity	
	Mean	SD	Mean	SD	Mean	SD
Austria	0.140	0.026	0.063	0.025	0.207	0.050
Belgium	0.240	0.154	0.187	0.177	0.307	0.220
Bulgaria	0.025	0.007	0.250	0.057	0.360	0.000
Croatia	0.160	0.044	0.007	0.006	0.137	0.038
Cyprus	0.130	–	0.040	–	0.030	–
Czech Republic	0.060	–	0.020	–	0.250	–
Denmark	0.110	–	0.040	–	0.130	–
Estonia	0.320	–	0.430	–	0.540	–
Finland	0.055	0.007	0.130	0.028	0.075	0.007
France	0.148	0.083	0.101	0.060	0.214	0.111
Germany	0.139	0.069	0.072	0.039	0.434	0.108
Greece	0.104	0.062	0.041	0.037	0.216	0.196
Iceland	0.060	–	0.010	–	0.160	–
Italy	0.046	0.024	0.238	0.093	0.036	0.013
Latvia	0.240	–	0.420	–	0.700	–
Luxembourg	0.430	–	0.500	–	0.430	–
Netherlands	0.135	0.049	0.108	0.116	0.508	0.181
Norway	0.124	0.046	0.084	0.032	0.184	0.093
Poland	0.033	0.022	0.005	0.008	0.032	0.015
Portugal	0.110	–	0.020	–	0.070	–
Romania	0.010	0.008	0.130	0.177	0.210	0.211
Slovakia	0.060	–	0.200	–	0.320	–
Slovenia	0.150	–	0.040	–	0.130	–
Spain	0.133	0.053	0.170	0.169	0.094	0.044
Sweden	0.180	0.066	0.093	0.040	0.260	0.100
Switzerland	0.303	0.049	0.310	0.108	0.512	0.076
Turkey	0.045	0.109	0.179	0.234	0.028	0.049
United Kingdom	0.152	0.105	0.060	0.059	0.537	0.077

*Source:* European Social Survey 1–4

With regard to diversity in origin, Table 6 shows the following. Bulgaria and Finland have the lowest value on this measure with a standard deviation of 0.00 and a range of 0.01. In contrast, Belgium and the United Kingdom have a standard deviation of 0.08 and 0.10 and a range of 0.30 and 0.39. In Hungary and Poland, the level of linguistic diversity varies the least across the NUTS level 1 regions of the countries in this sample (the standard deviations for both countries are 0.01 and the range is also 0.01). Quite different is the situation for Turkey and Spain where the standard deviations are 0.22 and 0.18 and the scores on linguistic diversity have a range 0.62 and 0.41. Finally, religious diversity also turns out to vary across the NUTS level 1 regions within countries. With respect to this kind of diversity, Bulgaria and Finland stand out with a standard deviation of 0.00 and a range of 0.00 and 0.01. On the other side there are countries like Romania and the Netherlands with a standard deviation of 0.18 and 0.16 and a range of 0.47 and 0.41.

This overview of how the measures of diversity constructed with the ESS data vary across regions within countries provide evidence for the conclusion that in some countries the different regions are far more similar to each other than in other countries. In practice, this means that in some countries people from different regions experience the same level of diversity, whereas in other countries this depends much more on where the person lives. With respect to the impact that diversity may have on people’s behavior, attitudes and opinions, these differences may indicate that studies relating national level diversity to individual level outcomes miss an important share of the variation in diversity across regions within countries.

## Effects of Diversity on Individual Sociality

### Trust and Government Redistribution

Empirical studies of diversity aim at investigating its effects on societal outcomes. The national level studies, relating national level diversity to social policies, do so by arguing that diversity affects individual preferences and their willingness to contribute to common goods (e.g. Alesina and Glaeser 2004). Single country studies mainly focused on interpersonal trust (e.g. Putnam 2007). Therefore, what these studies share is a general concern about whether or not diversity affects human sociality which governs social relationships between individuals (e.g. Henrich et al. 2004). In this section, three kinds of sociality are investigated that are close to the ones examined in previous studies, namely interpersonal trust, institutional trust, and support for government redistribution. The main aim of this part of the analysis is to examine whether regional diversity affects human sociality using international comparative data rather than single level data. To examine whether regional level indicators of diversity yield different individual outcomes than the ones at the national level the results for these indicators are compared. Research interest has been on the negative effects of societal diversity on sociality, for different reasons as the ethnic, linguistic and religious dimension of diversity affects individual outcomes through different mechanisms. Ethnic diversity is believed to hinder social sanctioning (Habyarimana et al. 2007), linguistic diversity hinders efficient communication (Anderson and Paskeviciute 2006), and religious diversity can lead to strong divisions between members and nonmembers (Yamagishi and Mifune 2008). Based on these mechanisms it is hypothesized that interpersonal trust, institutional trust, and support for government redistribution are negatively related to diversity and fractionalization. This general hypothesis closely resembles a general expectation across diversity studies, however it is acknowledged that other mechanisms may be at work that lead to contrasting hypotheses and that the effects of diversity differ at the national and the regional level (Burgoon et al. 2011).

### Measures and Method

#### Dependent Variables

The three independent variables examined here are measured in all four rounds of the ESS. *Interpersonal trust* is measured with the following item: “Please tell me on a score of 0–10, where 0 means you can’t be too careful and 10 means that most people can be trusted”. *Institutional trust* is measured by combining the responses to questions about people’s level of trust towards six different institutions. Respondents were asked to indicate their level of trust in “their country’s parliament”, “the legal system”, “the police”, “politicians”, “the European Parliament”, and “the United Nations” on a scale from 0 (no trust at all) to 10 (complete trust). The Cronbach’s alpha of the resulting scale is 0.87. *Support for government redistribution* is measured with the item: “The government should take measures to reduce differences in income levels” (1 = disagree strongly; 5 = agree strongly).

#### Independent Variables

The independent variables are the diversity in origin and linguistic and religious diversity indicators at the regional and the national level. The regional level indicators are the diversity measures reported in Appendix Table 10, which were constructed in the first part of this article, and the national level indicators are the diversity scores constructed by Alesina et al. (2003).

#### Control Variables

The data are a combination of round 1 through 4 of the ESS. To account for time trends, dummy variables indicating the ESS round are added to the model (Round 1 is the reference category). Furthermore, several background variables of the respondent are added to the model, namely gender (0 = male; 1 = female), age, and years of education. To control for national differences, Gross domestic product at purchasing power parity per capita (GDP) is added to the model.

The dependent variables are measured at the individual level. The independent and control variables are measured at three different levels of analysis (individual, regional, and national). To take this nested structure of the data into account a three-level multilevel model is constructed. The three dependent variables are investigated following the same steps. First a control model (Model 1) is calculated that functions as a point of reference to compare subsequent models. Then, three models are constructed investigating the effects of ethnic, linguistic, and religious heterogeneity at the regional level (Models 2a–c). And finally, three models are computed investigating ethnic, linguistic and religious heterogeneity at the national level (Models 3a–c). Changes in the fit of the model are assessed by computing the deviance using full information maximum likelihood (Snijders and Bosker 1999). The parameters in these models are estimated by the maximum likelihood method and the regression coefficients are tested by Wald tests (Goldstein 2003; Snijders 2003).

### Results of the Multilevel Analyses

The results of the multilevel analyses are reported in Tables 7, 8, and 9. Concentrating on the models with the control variables, the analyses show that the outcomes for the three kinds of sociality differ markedly. While people report higher levels of interpersonal trust and support for government redistribution in later rounds of the ESS compared to the first, their trust in institutions is lower in the later rounds of the ESS. Women report higher levels of interpersonal trust and they are more in favor of government redistribution, but gender does not explain variation in institutional trust. Interpersonal trust and support for government redistribution is negatively related to age, while institutional trust is not related to the age of respondents. Educational level is positively related to interpersonal trust and institutional trust and negatively related to support for government redistribution. Finally, while interpersonal trust and institutional trust are higher in more wealthy countries, GDP per capita is negatively related to support for government redistribution.

**Table 7:** Multilevel analysis of interpersonal trust

	(1)	(2a)	(2b)	(2c)	(3a)	(3b)	(3c)
*Regional level*							
Diversity in origin		−0.295 (0.377)					
Linguistic diversity			−0.169 (0.227)				
Religious diversity				0.316 (0.233)			
*Country level*							
Ethnic diversity					−1.197 (0.726)		
Linguistic diversity						−0.515 (0.692)	
Religious diversity							0.662 (0.586)
*Control variables*							
ESS Round 1 (reference)	–	–	–	–	–	–	–
ESS Round 2	0.038** (0.013)	0.038** (0.013)	0.038** (0.013)	0.038** (0.013)	0.038** (0.013)	0.038** (0.013)	0.038** (0.013)
ESS Round 3	0.111** (0.013)	0.111** (0.013)	0.111** (0.013)	0.112** (0.013)	0.111** (0.013)	0.111** (0.013)	0.111** (0.013)
ESS Round 4	0.129** (0.013)	0.129** (0.013)	0.129** (0.013)	0.129** (0.013)	0.129** (0.013)	0.129** (0.013)	0.129** (0.013)
Gender (1 = female)	0.071** (0.009)	0.071** (0.009)	0.071** (0.009)	0.071** (0.009)	0.071** (0.009)	0.071** (0.009)	0.071** (0.009)
Age	−0.003** (0.000)	−0.003** (0.000)	−0.003** (0.000)	−0.003** (0.000)	−0.003** (0.000)	−0.003** (0.000)	−0.003** (0.000)
Years of education	0.054** (0.001)	0.054** (0.001)	0.054** (0.001)	0.054** (0.001)	0.054** (0.001)	0.054** (0.001)	0.054** (0.001)
GDP per capita	0.609** (0.125)	0.625** (0.125)	0.613** (0.123)	0.604** (0.123)	0.591** (0.119)	0.630** (0.126)	0.628** (0.122)
Intercept	4.826 (0.130)	4.827 (0.129)	4.829 (0.129)	4.830 (0.129)	4.847 (0.125)	4.832 (0.129)	4.844 (0.128)
*Variance*							
Country level	0.465 (0.127)	0.462 (0.126)	0.459 (0.125)	0.456 (0.125)	0.425 (0.116)	0.457 (0.125)	0.443 (0.121)
Regional level	0.052 (0.009)	0.052 (0.009)	0.052 (0.009)	0.051 (0.008)	0.052 (0.009)	0.052 (0.009)	0.052 (0.009)
Individual level	3.136 (0.011)	3.136 (0.011)	3.136 (0.011)	3.136 (0.011)	3.136 (0.011)	3.136 (0.011)	3.136 (0.011)
Deviance^a^		0.616	0.557	1.844	2.613	0.546	1.248

*Sources:* European Social Survey 1–4, Alesina et al. (2003), and Eurostat

^a^Compared to Model 1

Individuals = 166,458; Regions = 123; Countries = 30

** *p* < 0.01; * *p* < 0.05

**Table 8:** Multilevel analysis of institutional trust

	(1)	(2a)	(2b)	(2c)	(3a)	(3b)	(3c)
*Regional level*							
Diversity in origin		0.811* (0.388)					
Linguistic diversity			0.933** (0.224)				
Religious diversity				0.314 (0.241)			
*Country level*					−1.202* (0.592)		
Ethnic diversity						−0.531 (0.570)	
Linguistic diversity							−0.873* (0.462)
Religious diversity							
*Control variables*							
ESS Round 1 (reference)	–	–	–	–	–	–	–
ESS Round 2	−0.178** (0.014)	−0.178** (0.014)	−0.178** (0.014)	−0.178** (0.014)	−0.178** (0.014)	−0.178** (0.014)	−0.178** (0.014)
ESS Round 3	−0.092** (0.014)	−0.092** (0.014)	−0.092** (0.014)	−0.092** (0.014)	−0.092** (0.014)	−0.092** (0.014)	−0.092** (0.014)
ESS Round 4	−0.140** (0.014)	−0.140** (0.014)	−0.140** (0.014)	−0.140** (0.014)	−0.140** (0.014)	−0.140** (0.014)	−0.140** (0.014)
Gender (1 = female)	−0.006 (0.009)	−0.006 (0.009)	−0.006 (0.009)	−0.006 (0.009)	−0.006 (0.009)	−0.006 (0.009)	−0.006 (0.009)
Age	0.000 (0.000)	0.000 (0.000)	0.000 (0.000)	0.000 (0.000)	0.000 (0.000)	0.000 (0.000)	0.000 (0.000)
Years of education	0.042** (0.001)	0.042** (0.001)	0.042** (0.001)	0.042** (0.001)	0.042** (0.001)	0.042** (0.001)	0.042** (0.001)
GDP per capita	0.520** (0.103)	0.476** (0.109)	0.496** (0.108)	0.514** (0.107)	0.500** (0.098)	0.542** (0.104)	0.500** (0.097)
Intercept	4.998 (0.107)	4.840 (0.131)	4.843 (0.130)	4.843 (0.131)	4.861 (0.126)	4.846 (0.130)	4.858 (0.129)
*Variance*							
Country level	0.302 (0.086)	0.334 (0.093)	0.347 (0.096)	0.333 (0.093)	0.267 (0.077)	0.295 (0.084)	0.263 (0.076)
Regional level	0.062 (0.010)	0.052 (0.009)	0.052 (0.009)	0.052 (0.009)	0.052 (0.009)	0.052 (0.009)	0.052 (0.009)
Individual level	3.113 (0.012)	3.113 (0.012)	3.113 (0.012)	3.113 (0.012)	3.113 (0.012)	3.113 (0.012)	3.113 (0.012)
Deviance^a^		3.900*	15.800**	1.500	3.800	0.800	3.300

*Sources:* European Social Survey 1–4, Alesina et al. (2003), and Eurostat

^a^Compared to Model 1

Individuals = 143,470; Regions = 123; Countries = 30

** *p* < 0.01; * *p* < 0.05

**Table 9:** Multilevel analysis of government redistribution

	(1)	(2a)	(2b)	(2c)	(3a)	(3b)	(3c)
*Regional level*							
Diversity in origin		−0.825** (0.226)					
Linguistic diversity			−0.219 (0.142)				
Religious diversity				−0.461** (0.128)			
*Country level*							
Ethnic diversity					0.002 (0.237)		
Linguistic diversity						−0.163 (0.215)	
Religious diversity							−0.372* (0.163)
*Control variables*							
ESS Round 1 (reference)	–	–	–	–	–	–	–
ESS Round 2	0.044** (0.007)	0.044** (0.007)	0.044** (0.007)	0.044** (0.007)	0.044** (0.007)	0.044** (0.007)	0.044** (0.007)
ESS Round 3	0.082** (0.007)	0.082** (0.007)	0.082** (0.007)	0.082** (0.007)	0.082** (0.007)	0.082** (0.007)	0.082** (0.007)
ESS Round 4	0.040** (0.007)	0.041** (0.007)	0.041** (0.007)	0.041** (0.007)	0.040** (0.007)	0.041** (0.007)	0.040** (0.007)
Gender (1 = female)	0.130** (0.005)	0.130** (0.005)	0.130** (0.005)	0.130** (0.005)	0.130** (0.005)	0.130** (0.005)	0.130** (0.005)
Age	−0.002** (0.000)	−0.002** (0.000)	−0.002** (0.000)	−0.002** (0.000)	−0.002** (0.000)	−0.002** (0.000)	−0.002** (0.000)
Years of education	−0.028** (0.001)	−0.028** (0.001)	−0.028** (0.001)	−0.028** (0.001)	−0.028** (0.001)	−0.028** (0.001)	−0.028** (0.001)
GDP per capita	−0.149** (0.039)	−0.103** (0.039)	−0.144** (0.040)	−0.138** (0.037)	−0.149** (0.040)	−0.143** (0.040)	−0.153** (0.036)
Intercept	3.633 (0.040)	3.634 (0.042)	3.627 (0.041)	3.628 (0.038)	3.633 (0.041)	3.634 (0.040)	3.626 (0.037)
*Variance*							
Country level	0.034 (0.012)	0.040 (0.013)	0.037 (0.012)	0.029 (0.010)	0.034 (0.012)	0.033 (0.011)	0.027 (0.010)
Regional level	0.026 (0.004)	0.022 (0.003)	0.025 (0.004)	0.024 (0.004)	0.026 (0.004)	0.026 (0.004)	0.026 (0.004)
Individual level	0.948 (0.003)	0.948 (0.003)	0.948 (0.003)	0.948 (0.003)	0.948 (0.003)	0.948 (0.003)	0.948 (0.003)
Deviance^a^		11.993**	2.297	12.187**	0.000	0.568	4.964*

*Sources:* European Social Survey 1–4, Alesina et al. (2003), and Eurostat

^a^Compared to Model 1

Individuals = 167,367; Regions = 123; Countries = 30

** *p* < 0.01; * *p* < 0.05

Turning to the models relating regional and national diversity to interpersonal trust, Table 7 shows that none of these indicators explains variation in this kind of sociality. All forms of regional and national level diversity, except religious diversity of the region, have a negative sign, but all these effects are not statistically significant.

Investigating institutional trust, Table 8 shows that it is positively related to diversity in origin and linguistic diversity at the regional level and negatively to ethnic diversity and religious diversity of the country. Nevertheless, focusing on how much the variables contribute to the fit of the model, it is evident that both effects at the regional level lead to a significant improvement (Deviance = 3.900; *p* < 0.05 for diversity in origin and Deviance = 15.800; *p* < 0.01 for linguistic diversity), while both diversity indicators at the national level do not lead to significant improvements of the model (Deviance = 3.800; n.s. for ethnic diversity and Deviance = 3.300; n.s. for religious diversity).

Table 9 shows the results for support for government redistribution. At the regional level, two of the three diversity measures are significantly related to this kind of sociality, namely diversity in origin (b = −0.825; *p* < 0.01) and religious diversity (b = −0.461; *p* < 0.01). In both these cases, the model fit improves significantly (Deviance = 11.993; *p* < 0.01 for diversity in origin and Deviance = 12.187; *p* < 0.01 for religious diversity). Besides that, support for government redistribution is significantly (at the 5 percent significance level) negative related to religious diversity (b = −0.372; *p* < 0.05; Deviance = 4.964; *p* < 0.05).

These results lead to the following conclusions. Interpersonal trust is neither affected by regional diversity nor by national level diversity. Comparing the outcomes for institutional trust and support for government redistribution suggests stronger effects of regional diversity than of national level diversity. However, the effects of regional diversity are not as straightforward as is suggested by a large share of the literature. While regional diversity is positively related to institutional trust, it is negatively related to support for government redistribution. This suggests that people are less willing to support the welfare state, but not because their trust in government institutions is in decline. As such, the outcomes challenge the belief that diversity affects societies in one direction and shows that contrasting effects are much more likely.

## Conclusions

Societal and scientific interest into the impact of migration requires constructing and using valid and informative indicators. The analyses provided here attempt to develop such indicators to measure the level of diversity of regions across European countries. An investigation of the extent to which the new indicators are in line with existing measures, leads to the conclusion that some groups may be underrepresented in the ESS. Nevertheless, overall, the similarity between the regional and national diversity indicators shows that they are strongly related suggests that surveys like the ESS are suitable to capture social structural diversity.

The next question addressed in this study concerns the level at which diversity is meaningfully measured. To date, two possibilities were available for investigating the effects of diversity, namely general measures at the national level across countries or specific measures at the neighborhood level within countries. This study provides a third possibility by constructing regional measures across countries. Comparing the effects of national diversity with regional diversity on different kinds of human sociality that have been the topic in previous studies –interpersonal trust, institutional trust, and support for government redistribution—shows that that regional level diversity is a stronger predictor of these individual outcomes than national level diversity. First, this result provides evidence for the idea that the regional level has a stronger impact on the preferences, attitudes, and behavior of individuals than the national level. As a consequence, the present study is critical of constructing multilevel models in which national level indicators are linked with individual outcomes, without paying attention to the meso level mediating or moderating the national and individual level. Secondly, this study has implications for studies relating diversity with individual preferences, attitudes, and behavior as it shows that diversity may generate contrasting results. Therefore, studies using only one dependent variable can lead to too bold conclusions about the direction of the impact of diversity. Including a wider range of response variables will help to overcome such bias. Although this may lead to contrasting results, it also leads to new questions concerning the mechanisms explaining the effects of diversity.

## Appendix

See Table 10.

**Table 10:** Number of respondents, missing values, and diversity per region, NUTS level 1

Country	Nuts 1 region	N	% Missing			Diversity		
			Origin	Language	Religion	Origin	Language	Religion
Austria	Ostösterreich	2,926	0.003	0.000	0.012	0.17	0.09	0.26
	Südösterreich	1,513	0.001	0.000	0.014	0.12	0.06	0.20
	Westösterreich	2,479	0.001	0.000	0.015	0.13	0.04	0.16
Belgium	Vlaams Gewest	4,451	0.000	0.000	0.002	0.11	0.10	0.17
	Région de Bruxelles-Capitale/Brussels Hoofdstedelijk Gewest	466	0.002	0.013	0.006	0.41	0.39	0.56
	Région Wallonne	2,318	0.000	0.002	0.004	0.20	0.07	0.19
Bulgaria	Severna i iztochna Bulgaria	1,927	0.000	0.000	0.006	0.03	0.29	0.36
	Yugozapadna i yuzhna centralna Bulgaria	1,703	0.000	0.000	0.009	0.02	0.21	0.36
Croatia	Sjeverozapadna Hrvatska	413	0.002	0.002	0.017	0.14	0.00	0.11
	Sredisnja i Istocna (Panonska) Hrvatska	420	0.000	0.000	0.007	0.21	0.01	0.18
	Jadranska Hrvatska	560	0.000	0.000	0.007	0.13	0.01	0.12
Cyprus	Cyprus	2,210	0.000	0.000	0.011	0.13	0.04	0.03
Czech Republic	Czech Republic	6,400	0.007	0.003	0.013	0.06	0.02	0.25
Denmark	Denmark	6,108	0.003	0.019	0.007	0.11	0.04	0.13
Estonia	Estonia	5,167	0.007	0.006	0.005	0.32	0.43	0.54
Finland	Manner-Suomi	6,259	0.000	0.004	0.000	0.05	0.11	0.07
	Åland	1,854	0.000	0.005	0.005	0.06	0.15	0.08
France	Île de France	1,109	0.000	0.005	0.007	0.31	0.20	0.46
	Bassin Parisien Est	583	0.000	0.007	0.005	0.10	0.08	0.09
	Bassin Parisien Ouest	714	0.000	0.000	0.007	0.10	0.12	0.12
	Nord—Pas-de-Calais	546	0.000	0.004	0.004	0.09	0.07	0.20
	Est	734	0.000	0.037	0.003	0.13	0.20	0.27
	Ouest	1,014	0.000	0.001	0.001	0.07	0.04	0.13
	Sud-Ouest	925	0.000	0.002	0.001	0.11	0.05	0.18
	Sud-Est	952	0.000	0.001	0.002	0.16	0.07	0.25
	Méditerranée	791	0.000	0.001	0.005	0.26	0.08	0.23
Germany	Schleswig–Holstein	295	0.003	0.007	0.003	0.13	0.07	0.29
	Hamburg	207	0.000	0.000	0.000	0.22	0.13	0.45
	Niedersachsen	854	0.001	0.016	0.004	0.15	0.06	0.43
	Bremen	82	0.000	0.000	0.012	0.13	0.07	0.28
	Nordrhein-Westfalen	2,028	0.000	0.013	0.007	0.20	0.11	0.58
	Hessen	614	0.000	0.008	0.002	0.25	0.13	0.54
	Rheinland-Pfalz	440	0.000	0.007	0.007	0.15	0.07	0.53
	Baden-Württemberg	1,112	0.001	0.001	0.008	0.22	0.11	0.59
	Bayern	1,356	0.000	0.018	0.005	0.19	0.10	0.46
	Saarland	107	0.000	0.000	0.000	0.18	0.07	0.43
	Berlin	698	0.001	0.006	0.004	0.15	0.10	0.58
	Brandenburg	647	0.000	0.006	0.002	0.05	0.05	0.35
	Mecklenburg-Vorpommern	535	0.000	0.002	0.004	0.06	0.03	0.38
	Sachsen	1,123	0.000	0.021	0.002	0.06	0.03	0.31
	Sachsen-Anhalt	693	0.000	0.010	0.006	0.04	0.01	0.30
	Thüringen	665	0.000	0.012	0.006	0.05	0.01	0.44
Greece	Voreia Ellada	2,400	0.000	0.000	0.000	0.13	0.08	0.07
	Kentriki Ellada	1,496	0.004	0.000	0.001	0.11	0.04	0.05
	Attiki	2,457	0.004	0.000	0.001	0.20	0.09	0.07
	Nisia Aigaiou, Kriti	691	0.003	0.000	0.004	0.15	0.06	0.07
Hungary	Közép-Magyarország	1,511	0.001	0.001	0.005	0.07	0.00	0.44
	Dunántúl	2,122	0.000	0.000	0.003	0.04	0.02	0.31
	Alföld és Észak	2,612	0.000	0.000	0.005	0.03	0.00	0.50
Iceland	Iceland	579	0.014	0.014	0.016	0.06	0.01	0.16
Ireland	Ireland	6,132	0.002	0.024	0.007	0.16	0.05	0.10
Italy	Nord Ovest	591	0.000	0.000	0.007	0.04	0.16	0.05
	Nord Est	468	0.000	0.000	0.006	0.08	0.28	0.03
	Centro (IT)	521	0.000	0.000	0.006	0.06	0.12	0.03
	Sud (IT)	799	0.000	0.000	0.005	0.03	0.29	0.02
	Isole (IT)	357	0.000	0.000	0.008	0.02	0.34	0.05
Latvia	Latvia	1,980	0.000	0.000	0.007	0.24	0.42	0.70
Luxembourg	Luxembourg (Grand-Duché)	3,187	0.001	0.002	0.010	0.43	0.50	0.43
Netherlands	Noord-Nederland	878	0.000	0.000	0.001	0.08	0.28	0.49
	Oost-Nederland	1,718	0.000	0.000	0.001	0.13	0.04	0.61
	West-Nederland	3,635	0.000	0.000	0.002	0.20	0.07	0.67
	Zuid-Nederland	1,681	0.000	0.000	0.001	0.13	0.04	0.26
Norway	Oslo og Akershus	1,530	0.001	0.006	0.001	0.21	0.14	0.30
	Hedmark og Oppland	572	0.000	0.007	0.000	0.08	0.04	0.09
	Sør-Østlandet	1,291	0.000	0.002	0.001	0.15	0.07	0.32
	Agder og Rogaland	1,000	0.000	0.003	0.000	0.14	0.08	0.20
	Vestlandet	1,292	0.001	0.002	0.002	0.10	0.08	0.13
	Trøndelag	672	0.000	0.007	0.000	0.11	0.11	0.14
	Nord-Norge	738	0.000	0.004	0.000	0.08	0.07	0.11
Poland	Centralny	1,580	0.001	0.000	0.003	0.01	0.00	0.02
	Poludniowy	1,428	0.001	0.000	0.008	0.02	0.01	0.02
	Wschodni	1,393	0.001	0.001	0.001	0.02	0.02	0.06
	Pólnocno-Zachodni	874	0.000	0.000	0.002	0.04	0.00	0.03
	Poludniowo-Zachodni	652	0.002	0.000	0.003	0.07	0.00	0.03
	Pólnocny	1,239	0.001	0.000	0.006	0.04	0.00	0.03
Portugal	Continente (PT)	8,152	0.002	0.005	0.002	0.11	0.02	0.07
Romania	Macroregiunea unu	502	0.024	0.000	0.012	0.00	0.39	0.51
	Macroregiunea doi	647	0.017	0.000	0.005	0.02	0.04	0.09
	Macroregiunea trei	561	0.029	0.002	0.004	0.01	0.00	0.04
	Macroregiunea patru	436	0.016	0.005	0.000	0.01	0.09	0.20
Slovakia	Slovakia	5,061	0.002	0.012	0.010	0.06	0.20	0.32
Slovenia	Slovenia	5,723	0.006	0.002	0.083	0.15	0.04	0.13
Spain	Noroeste	1,143	0.000	0.000	0.003	0.09	0.38	0.04
	Noreste	735	0.000	0.000	0.005	0.11	0.13	0.10
	Comunidad de Madrid	971	0.000	0.000	0.010	0.22	0.06	0.11
	Centro (ES)	998	0.001	0.000	0.007	0.07	0.03	0.05
	Este	2,140	0.000	0.000	0.007	0.17	0.44	0.17
	Sur	1,574	0.000	0.000	0.004	0.11	0.04	0.08
	Canarias (ES)	283	0.000	0.000	0.004	0.16	0.11	0.11
Sweden	Östra Sverige	2,728	0.001	0.000	0.003	0.24	0.13	0.36
	Södra Sverige	3,412	0.000	0.001	0.002	0.19	0.10	0.26
	Norra Sverige	1,564	0.000	0.000	0.001	0.11	0.05	0.16
Switzerland	Région lémanique	1,465	0.000	0.001	0.002	0.39	0.39	0.54
	Espace Mittelland	1,642	0.000	0.000	0.002	0.24	0.49	0.50
	Nordwestschweiz	1,317	0.000	0.002	0.004	0.30	0.27	0.58
	Zürich	1,187	0.000	0.001	0.002	0.30	0.26	0.51
	Ostschweiz	1,437	0.000	0.000	0.004	0.31	0.24	0.57
	Zentralschweiz	544	0.000	0.000	0.007	0.28	0.21	0.37
	Ticino	212	0.000	0.000	0.000	0.40	0.35	0.16
Turkey	Istanbul	533	0.000	0.000	0.004	0.01	0.08	0.05
	Bati Marmara	88	0.000	0.000	0.000	0.00	0.02	0.02
	Ege	277	0.000	0.000	0.000	0.05	0.01	0.00
	Dogu Marmara	280	0.000	0.000	0.004	0.08	0.04	0.10
	Bati Anadolu	214	0.000	0.000	0.000	0.01	0.04	0.01
	Akdeniz	314	0.000	0.000	0.000	0.01	0.06	0.00
	Orta Anadolu	67	0.000	0.000	0.000	0.00	0.06	0.00
	Bati Karadeniz	80	0.000	0.000	0.000	0.02	0.00	0.02
	Dogu Karadeniz	31	0.000	0.000	0.000	0.00	0.00	0.00
	Kuzeydogu Anadolu	68	0.000	0.000	0.000	0.00	0.55	0.00
	Ortadogu Anadolu	129	0.000	0.000	0.000	0.00	0.50	0.00
	Güneydogu Anadolu	335	0.000	0.000	0.000	0.01	0.62	0.00
United Kingdom	North East (ENGLAND)	411	0.000	0.000	0.000	0.06	0.02	0.54
	North West (ENGLAND)	1,035	0.001	0.004	0.002	0.13	0.06	0.59
	Yorkshire and The Humber	749	0.000	0.004	0.001	0.14	0.06	0.57
	East Midlands (ENGLAND)	666	0.000	0.000	0.002	0.10	0.02	0.48
	West Midlands (ENGLAND)	727	0.000	0.008	0.000	0.18	0.09	0.56
	South West (ENGLAND)	783	0.000	0.001	0.000	0.11	0.02	0.43
	Eastern	843	0.000	0.002	0.002	0.21	0.05	0.51
	London	760	0.001	0.020	0.003	0.45	0.23	0.74
	South East	1,180	0.001	0.010	0.001	0.18	0.04	0.51
	Wales	510	0.000	0.000	0.000	0.06	0.08	0.51
	Scotland	752	0.001	0.003	0.003	0.11	0.04	0.52
	Northern Ireland	279	0.000	0.000	0.000	0.09	0.01	0.49
Total	171,619		0.002	0.004	0.008	0.12	0.12	0.26

*Sources:* European Social Survey 1–4
